# Baseline Prediction of Combination Therapy Outcome in Hepatitis C Virus 1b Infected Patients by Discriminant Analysis Using Viral and Host Factors

**DOI:** 10.1371/journal.pone.0014132

**Published:** 2010-11-30

**Authors:** Verónica Saludes, Maria Alma Bracho, Oliver Valero, Mercè Ardèvol, Ramón Planas, Fernando González-Candelas, Vicente Ausina, Elisa Martró

**Affiliations:** 1 Microbiology Service, Fundació Institut d'Investigació en Ciències de la Salut Germans Trias i Pujol, Hospital Universitari Germans Trias i Pujol, Universitat Autònoma de Barcelona, Badalona, Spain; 2 CIBER Epidemiología y Salud Pública (CIBERESP), Barcelona, Spain; 3 Unidad Mixta Genómica y Salud, Centro Superior de Investigación en Salud Pública - Universitat de València (CSISP-UV)/Instituto Cavanilles, Valencia, Spain; 4 Statistics Service, Universitat Autònoma de Barcelona, Cerdanyola, Spain; 5 Hospital Pharmacy, Hospital Universitari Germans Trias i Pujol, Badalona, Spain; 6 Liver Unit, Hospital Universitari Germans Trias i Pujol, Badalona, Spain; 7 CIBER Enfermedades Hepáticas y Digestivas (CIBEREHD), Barcelona, Spain; 8 CIBER Enfermedades Respiratorias (CIBERES), Bunyola, Spain; St. Louis University, United States of America

## Abstract

**Background:**

Current treatment of chronic hepatitis C virus (HCV) infection has limited efficacy −especially among genotype 1 infected patients−, is costly, and involves severe side effects. Thus, predicting non-response is of major interest for both patient wellbeing and health care expense. At present, treatment cannot be individualized on the basis of any baseline predictor of response. We aimed to identify pre-treatment clinical and virological parameters associated with treatment failure, as well as to assess whether therapy outcome could be predicted at baseline.

**Methodology:**

Forty-three HCV subtype 1b (HCV-1b) chronically infected patients treated with pegylated-interferon alpha plus ribavirin were retrospectively studied (21 responders and 22 non-responders). Host (gender, age, weight, transaminase levels, fibrosis stage, and source of infection) and viral-related factors (viral load, and genetic variability in the E1–E2 and Core regions) were assessed. Logistic regression and discriminant analyses were used to develop predictive models. A “leave-one-out” cross-validation method was used to assess the reliability of the discriminant models.

**Principal Findings:**

Lower alanine transaminase levels (ALT, *p* = 0.009), a higher number of quasispecies variants in the E1–E2 region (number of haplotypes, nHap_E1–E2) (*p* = 0.003), and the absence of both amino acid arginine at position 70 and leucine at position 91 in the Core region (*p* = 0.039) were significantly associated with treatment failure. Therapy outcome was most accurately predicted by discriminant analysis (90.5% sensitivity and 95.5% specificity, 85.7% sensitivity and 81.8% specificity after cross-validation); the most significant variables included in the predictive model were the Core amino acid pattern, the nHap_E1–E2, and gamma-glutamyl transferase and ALT levels.

**Conclusions and Significance:**

Discriminant analysis has been shown as a useful tool to predict treatment outcome using baseline HCV genetic variability and host characteristics. The discriminant models obtained in this study led to accurate predictions in our population of Spanish HCV-1b treatment naïve patients.

## Introduction

Hepatitis C virus (HCV), with an estimated 170 million people infected worldwide, is the major causative agent of chronic liver disease, cirrhosis and hepatocellular carcinoma [1]. HCV is an enveloped positive single-stranded RNA virus and its genome exhibits significant genetic variability, which has been used to classify the virus into six major genotypes and a number of subtypes [2]. Furthermore, a high replication rate and the lack of proofreading activity of the viral RNA-dependent RNA polymerase generate a dynamic mosaic of closely related variants, usually referred to as quasispecies, within an infected individual. This phenomenon allows chronic infection establishment and may also have important implications in pathogenicity and resistance to antiviral drugs [3].

Pegylated-interferon alpha (PegIFN-α) and ribavirin (RBV) combination therapy constitutes the current standard of care for chronic hepatitis C treatment [4]. Despite recent advances in the development of “specifically targeted antiviral therapy for hepatitis C” (STAT-C) compounds, with protease inhibitors in phase III studies, possible future treatment regimens are likely to continue including these drugs in order to prevent HCV resistance [5]. Combination treatment is costly, requires long-term follow-up, and involves severe side effects. Furthermore, HCV genotype 1 infected patients fail to achieve a sustained virological response (SVR) in about 40–50% of the cases [6], [7]. Genotype 1 is the most common genotype worldwide; HCV subtype 1b (HCV-1b) is the most prevalent in Southern and Eastern Europe, Japan and other countries [8], [9] and is associated with a higher risk for hepatocellular carcinoma development [10].

A number of host-related factors have been associated with a lower likelihood of response to treatment, such as African-American ancestry, advanced liver fibrosis or cirrhosis, older age, male gender, obesity, transaminase levels, and host genetic polymorphisms [6], [7], [11]–[18]. Among the later, the rs12979860 polymorphism near the *IL28B* gene is the strongest predictive factor of SVR identified so far [14]; however, European-American patients not having the most favourable genotype (C/C) still have approximately 40% chance of responding to therapy (negative predictive value (NPV) around 60%). With regards to baseline virological factors, high viral loads, high levels of genetic variability within the E1–E2 and NS5A regions, as well as mutations in the so-called interferon sensitivity determining region (ISDR) and Core regions, have been related to therapeutic failure. Nevertheless, such findings have not been found in other studies and remain controversial [11], [19].

As predicting non-response prior to treatment is of major interest for both patient wellbeing and health care expense, several predictive models with variable accuracy have been proposed for HCV-1, such as those based in clinical variables in combination with viral load [20] or the ISDR mutant [21], as well as amino acid covariance in the full viral coding region [22]. However, according to present guidelines for patient management, individual treatment outcomes can only be precisely predicted once treatment is initiated on the basis of viral kinetics; a ≥2-Log(HCV-RNA) decline at week 12 (early virological response) is the most robust approach for identifying non-responder patients (NPV, 97–100%) and thus constitutes the earliest treatment-stopping rule [4].

The goal of this study was to identify pre-treatment clinical and virological parameters associated with treatment failure, as well as to assess whether therapy outcome could be predicted at baseline by means of comprehensive statistical methods in HCV-1b treatment naïve patients. Our results show that discriminant analysis could be a useful tool to predict treatment outcome using both baseline HCV genetic variability and host characteristics. The discriminant models obtained in this study lead to accurate predictions in our population of Spanish HCV-1b patients.

## Results

### Treatment response groups and adherence

Forty-three white Spanish patients met the inclusion criteria, 21 being responders and 22 non-responders. All patients were on treatment for the complete expected time and adherence to both drugs was overall >80%. No significant differences were observed between groups: 20 (95.2%) and 22 (100%) responders and non-responders had a good adherence to PegIFN-α, respectively (*p* = 0.488), and these proportions were 17 (80.9%) and 20 (90.9%) for RBV (*p* = 0.412).

### Baseline clinical variables associated with treatment outcome

Baseline clinical characteristics of patients according to treatment outcome and bivariate analyses results are shown in **Table 1**. Responder and non-responder groups were comparable in terms of gender, age, source of infection, and liver fibrosis stage (liver biopsy was not performed in 37.2% of the patients). Regarding body weight, one outlier was identified corresponding to a responder patient with 101.40 Kg, and differences between groups became significant when this patient was excluded (70.79±8.35 vs. 78.51±14.96 Kg in responder and non-responder groups, respectively *p* = 0.048). The alanine transaminase (ALT) quotient was significantly higher in responders than in non-responders (*p* = 0.009). Conversely, the gamma-glutamyl transferase (GGT) quotient tended to be higher in the non-responder group; two outliers were identified, which corresponded to two responder patients, and the GGT quotient was significantly higher in the non-responder group when these outliers were excluded (median, 0.58 and 1.07 in responders and non-responders, respectively, *p* = 0.033). The aspartate transaminase (AST) quotient was similar in both groups.

**Table 1:** Baseline clinical features of study patients according to treatment response group.

Patient characteristic		Responders (*n* = 21)	Non-responders (*n* = 22)	*p-*value
Male gender, *n* (%)		9 (42.9)	14 (63.6)	0.172
Age a		47.52±9.66	48.55±12.39	0.764
Weight (Kg) a		72.24±10.53	78.51±14.96	0.122
Source of infection, *n* (%)	Blood transfusion	6 (28.6)	10 (45.5)	1.000
	Non blood transfusion	2 (9.5)	2 (9.1)	
	Unknown	13 (61.9)	10 (45.5)	
Liver fibrosis stage, *n* (%)	F0-2	11 (52.3)	10 (45.5)	0.648
	F3-4	2 (9.5)	4 (18.2)	
	Unknown	8 (38.1)	8 (36.4)	
ALT quotient (×ULN) b		2.51 (1.32–4.15)	1.53 (0.15–4.90)	0.009
AST quotient (×ULN) a		1.74±0.50	1.54±0.74	0.328
GGT quotient (×ULN) b		0.58 (0.22–1.80)	1.12 (0.18–2.50)	0.111

ALT, alanine transaminase; AST, aspartate transaminase; GGT, gamma-glutamyl transferase; ×ULN, factor times upper limit of normal used in our center for males and females: 41 and 31 U/L for ALT, 37 and 31 for AST, and 85 and 50 for GGT, respectively;

a Data presented as mean ± SD, Student's *t* test;

b Data presented as median (range), Mann-Whitney *U* test.

### Baseline virological variables associated with treatment outcome

#### HCV viral load

Viral load did not differ significantly between groups (*p* = 0.210), with a mean value of 5.75±0.86 Log(IU/ml) in responders, and 6.03±0.58 Log(IU/ml) in non-responders.

#### E1–E2 genetic variability estimates

The median number of clones sequenced per patient was 22 (range, 20–33) in responders and 23 (range, 20–27) in non-responders (*p* = 0.291), yielding a total of 993 sequences. Genetic variability estimates according to treatment outcome and genomic region are shown in **Table 2**. Although non-responder patients tended to have higher values than those with SVR for most E1–E2 genetic variability estimates, the number of quasispecies variants (number of haplotypes, nHap) was the only factor that significantly differed between groups (*p* = 0.003). Regarding the hypervariable regions (HVR), the HVR-1 showed the highest values for all parameters; the nHap and the number of synonymous substitutions per synonymous site (Ks) in this region were marginally significant, both being higher in non-responders.

**Table 2:** Summary of viral genetic variability estimates according to genomic region.*

	E1–E2 region			HVR-1 subregion			HVR-2 subregion			HVR-3 subregion		
Estimator	Responders (*n* = 21)	Non-responders (*n* = 22)	*p-*value	Responders (*n* = 21)	Non-responders (*n* = 22)	*p-*value	Responders (*n* = 21)	Non-responders (*n* = 22)	*p-*value	Responders (*n* = 21)	Non-responders (*n* = 22)	*p-*value
**S**	60.9±42.9	68.0±26.7	0.525	16.0 (1–48)	17.0 (0–46)	0.319	3.0 (0–13)	4.0 (1–10)	0.366	13.7±10.2	13.1±6.9	0.817
**η**	48.0 (9–154)	63.5 (29–142)	0.290	17.0 (1–64)	18.0 (0–59)	0.458	3.0 (0–13)	4.0 (1–11)	0.282	11.0 (2–42)	12.0 (5–34)	0.981
**nHap**	17 (5–25)	22 (11–27)	0.003	11 (2–17)	12 (1–18)	0.090	4 (1–12)	5 (2–10)	0.281	9.5±5.4	10.8±3.4	0.384
**π**	0.019 (0.002–0.089)	0.024 (0.005–0.077)	0.496	0.043 (0.001–0.261)	0.063 (0.000–0.256)	0.716	0.035 (0.000–0.186)	0.032 (0.003–0.176)	0.734	0.019 (0.001–0.132)	0.021 (0.005–0.099)	0.923
**Ka**	0.013 (0.000–0.063)	0.014 (0.001–0.060)	0.827	0.052 (0.000–0.294)	0.057 (0.000–0.279)	0.903	0.030 (0.000–0.195)	0.028 (0.000–0.221)	0.961	0.016 (0.000–0.084)	0.009 (0.001–0.084)	0.536
**Ks**	0.057±0.050	0.064±0.035	0.609	0.070±0.060	0.112±0.079	0.064	0.041 (0.000–0.591)	0.048 (0.000–0.190)	0.864	0.040 (0.004–0.322)	0.056 (0.014–0.162)	0.610

*Nucleotide positions corresponding to the H77 reference sequence (GenBank accession number AF009606): E1–E2 region, 1322–1853; HVR-1, 1491–1571; HVR-2, 1761–1787; HVR-3, 1632–1739.

S, total number of polymorphic sites; η, total number of mutations; nHap, number of haplotypes; π, nucleotide diversity corrected by Jukes-Cantor method; Ka, number of nonsynonymous substitutions per nonsynonymous site; Ks, number of synonymous substitutions per synonymous site; Data are expressed as mean ± SD, Student's *t* test or median (range), Mann-Whitney *U* test.

#### Phylogenetic analysis of the E1–E2 region

Differentiated clusters corresponding to responder and non-responder patients were not observed (**Figure S1**). Patients 1746 and 3468 appeared to be closely epidemiologically related since they shared a monophyletic clade with a 100% bootstrap support. In this clade, sequences from patient 1746 were a subgroup of those obtained from patient 3468, thus pointing to a source-recipient relationship. Patients 1634 and 3030, and 587 and 1313 might also be epidemiologically related, as inferred from the highly supported clade encompassing sequences from both patients in each group (100 and 90% bootstrap values, respectively), but no source-recipient relationship could be inferred.

#### Analysis of amino acid composition of the E1–E2 region

None of the nine amino acid positions initially identified by VESPA analysis showed a significantly different composition between responders and non-responders after the false discovery rate correction was applied (data not shown).

#### Analysis of amino acid composition of the Core region

VESPA analysis did not identify any amino acid position that differed between groups, although a polymorphism at position 70 was detected. On the other hand, when pairs of observed polymorphisms were subjected to bivariate analysis, the absence of both amino acids arginine (R) at position 70 and leucine (L) at position 91 was observed in 5 of 21 responder patients (23.8%) and in 12 of 22 non-responders (54.5%), (*p* = 0.039). R70 was substituted either by glutamine (Q) or histidine (H), and L91 mostly by methionine (M) and by cysteine (C) in one case. Since phylogenetic analysis showed that patients with this amino acid pattern did not group within the same cluster, the observed association was not attributed to sharing a common ancestry. This phylogenetic analysis provided similar evidence regarding to epidemiological relationships described for the E1–E2 region (data not shown).

### Prediction of the treatment outcome according to baseline host and virological variables

#### Logistic regression analysis

**Figure 1:**
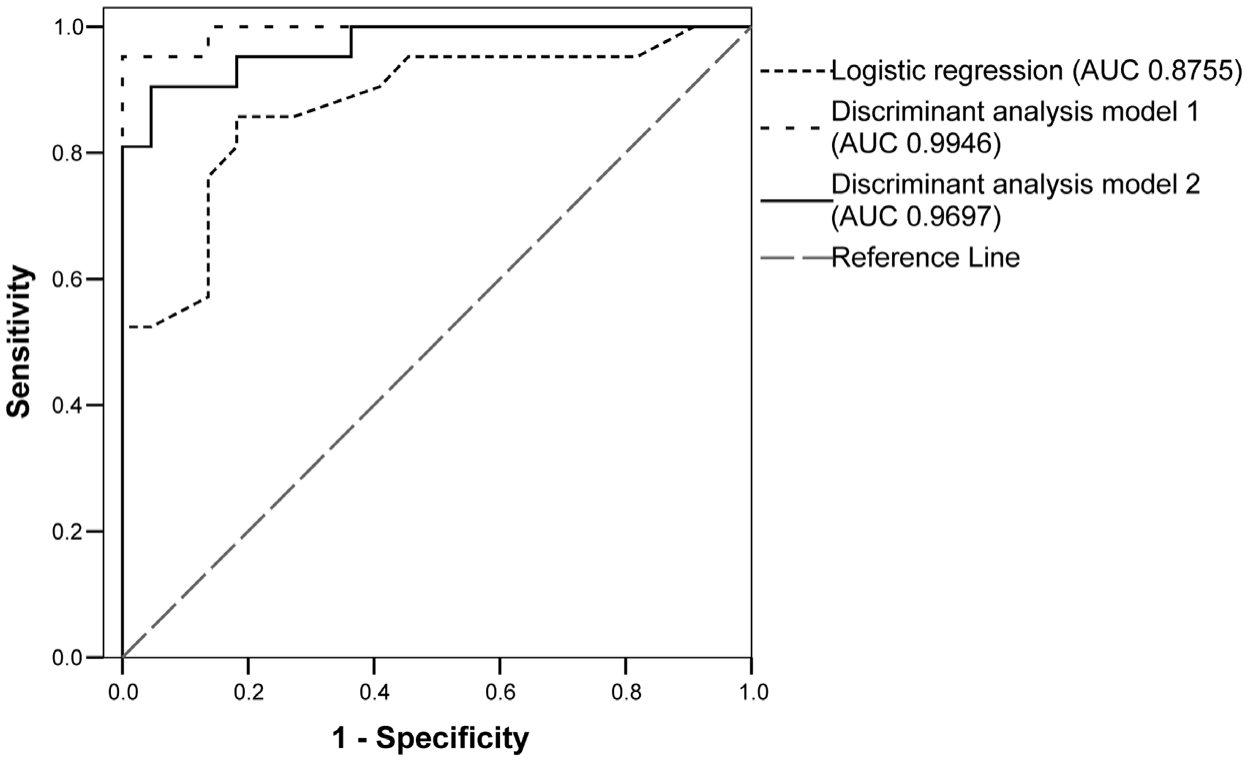
Receiver operating characteristic (ROC) curves for the multivariate logistic regression analysis and discriminant analysis models. AUC, area under the ROC curve; Sensitivity, proportion of responders which are correctly identified; Specificity, proportion of non-responders which are correctly identified. Variables included in the models in decreasing order of significance: logistic regression model, Core amino acid pattern and nHap_E1–E2; discriminant analysis model 1, Core amino acid pattern, nHap_E1–E2, Sqrt(GGT quotient), Sqrt(ALT quotient), Log(viral load), Sqrt(S_HVR-2), body weight, and Log(Ks_E1–E2); discriminant analysis model 2, Core amino acid pattern, nHap_E1–E2, Sqrt(GGT quotient), Sqrt(ALT quotient), nHap_HVR-1, and Sqrt(Ks_HVR-1), and body weight.

Variables showing a *p*-value <0.2 in the bivariate analyses (gender, Sqrt(ALT quotient), Sqrt(GGT quotient), weight, Core amino acid pattern, nHap_E1–E2, Log(Ks_E1–E2), nHap_HVR-1, and Sqrt(Ks_HVR-1)) were initially considered; the nHap_E1–E2 and the Core amino acid pattern persisted in the final model (**Text S1**), with an odds ratio (OR) of 1.47 (95% confidence interval, CI_95%_ = [1.16–1.87]) and 25.47 (CI_95%_ = [2.52–257.74]), respectively. Thus, the absence of amino acids R70 and L91 and a higher nHap_E1–E2 significantly increased the risk for treatment failure. An area under the curve (AUC) of 0.8755 was obtained in the receiver operating characteristic (ROC) curve (**Figure 1**), and selecting a 0.500 cut-off yielded a sensitivity and positive predictive value (PPV) of 81.0%, and a specificity and NPV of 81.8%.

#### Discriminant analysis

Two discriminant functions were obtained (**Text S1**) and cross-validated to assess how the results obtained would generalize to an independent but similar data set. Variables that persisted in Model 1 were: Core amino acid pattern, nHap_E1–E2, Sqrt(GGT quotient), Sqrt(ALT quotient), Log(viral load), Sqrt(total number of polymorphic sites in the HVR-2, S_HVR-2), body weight, and Log(Ks_E1–E2), in decreasing order of significance. The ROC curve obtained had an AUC of 0.9946 (**Figure 1**). This model yielded a 95.2% sensitivity and a 100% specificity (**Table 3**); however, sensitivity decreased to 76.2% and specificity to 72.7% after cross-validation. Therefore, we developed model 2 including the Core amino acid pattern, nHap_E1–E2, Sqrt(GGT quotient), Sqrt(ALT quotient), nHap_HVR-1, and Sqrt(Ks_HVR-1), and body weight, in decreasing order of significance. The AUC of the corresponding ROC curve was 0.9697 (**Figure 1**). Treatment outcome was predicted with 90.5% sensitivity and 95.5% specificity (cut-off, 0.550), and these values remained high after the cross-validation (85.7% and 81.8%, respectively). Besides, the model could be optimized to correctly identify most responder patients by choosing a cut-off of 0.900, so that treatment is not denied to individuals that are likely to respond (NPV, 93.3% after cross-validation). Sensitivity, specificity, NPV and PPV for different cut-offs are shown in **Table 3**. According to cross-validation, in an independent but similar data set, treatment could be omitted in 63.6 to 81.8% of the non-responder patients while most patients likely to respond would be identified and treated.

**Table 3:** Sensitivity, specificity, and predictive values for the discriminant models obtained.

	AUC	Cut-off	Sensitivity, % (cross-validated)	Specificity, % (cross-validated)	NPV, % (cross-validated)	PPV, % (cross-validated)
**Model 1**	0.9946	0.500	95.2 (76.2)	100 (72.7)	95.7 (76.2)	100 (72.7)
**Model 2**	0.9697	0.550	90.5 (85.7)	95.5 (81.8)	95.0 (81.8)	91.3 (85.7)
		0.900	95.2 (95.2)	68.2 (63.6)	93.8 (93.3)	74.1 (71.4)

AUC, area under the receiver operating characteristic curve; PPV, positive predictive value; NPV, negative predictive value.

## Discussion

As combination treatment failure occurs in about half of all patients with chronic hepatitis C infected by genotype 1 [6], [7], prediction of treatment outcome at baseline would be highly beneficial. Although several factors have been identified as predictors of treatment outcome, none of them can provide a reliable individualized prediction when used independently. Based on our results in Spanish patients infected with HCV-1b, we propose the use of discriminant statistical models based on host and viral characteristics to provide an aggregate prediction of the treatment outcome at baseline.

Among the host-related factors studied baseline ALT levels, which are an indicator of liver damage, were significantly higher in responder patients than in non-responders (*p = *0.009), as previously reported [11], [12]. Conversely, the GGT quotient tended to be higher in the non-responder group in agreement with other studies [12], [23]; higher GGT levels have been related to advanced fibrosis, steatosis and insuline resistance, which are more common among non-responders [24]. The body weight tended to be higher in non-responder patients; in fact, it has been suggested that obese subjects have an increased expression of the IFN-α signalling inhibitor factor SOCS-3 [25]. Some of the host factors that have previously been associated with treatment failure, such as male gender, advanced age, advanced liver fibrosis stage and cirrhosis [6], [7], [13] did not reach statistical significance in our study probably due to a limited sample size, especially regarding the liver biopsy, which was not performed in 37.2% of patients.

In relation to virus-related factors, HCV baseline viral load has been suggested as a predictor of SVR, but several cut-offs have been proposed [24]. In our study, average viral loads were higher in non-responders but differences were not significant. Additionally, several studies have reported an association between the level of variability in the HCV genome at baseline and treatment outcome. Envelope glycoprotein coding regions are highly variable; the HVR-1, which is the most variable region in the whole genome, is targeted by host neutralizing antibodies and plays a role in immune escape [26]. While the variability in this region has also been associated with treatment outcome [27]–[32], discrepancies on this matter have been noted probably due to the different treatment regimens, the different genetic variability estimates employed, and limitations in statistical analyses [33]–[35]. While our results show that treatment outcome was not related to the presence of a common evolutionary origin, in general terms, the E1–E2 genetic variability estimators suggested that a high heterogeneity in the baseline viral population could be involved in combination therapy failure, either through the pre-existence or the generation of drug-resistant viral variants. A higher number of quasispecies variants in the E1–E2 region (nHap_E1–E2) was significantly associated with treatment failure (*p* = 0.003). Additionally, when the analysis focussed on the HVR-1 subregion, nHap and Ks were marginally significant with higher values in the non-responder group. Although significant differences between groups at the amino acid level were not found, synonymous substitutions may have an effect on the secondary structure of the genomic RNA, which is an important selection target [36].

Pre-treatment Core amino acid substitutions at positions 70 (R by Q) and/or 91 (L by M) have been described as useful independent predictors of treatment failure in Japanese HCV-1b infected patients [37]. Similarly, our results show an association between the absence of both R70 and L91 amino acids and treatment failure (*p* = 0.039). Although it has been suggested that the Core protein may inhibit the transcription of antiviral genes induced by IFN-α [38], further studies are needed to clarify the role of the observed amino acid substitutions in treatment failure.

Since factors that significantly differed between groups in the bivariate analyses were not completely reliable in predicting treatment outcome when used independently, we developed predictive models that included a combination of variables. The logistic regression analysis identified the nHap_E1–E2 (OR = 1.47) and the Core amino acid pattern (OR = 25.47) as independent risk factors for treatment failure. However, predictive models obtained by discriminant analysis including additional variables showed better AUC values and more accurate predictions in our study population (90.5–95.2% sensitivity and 95.5–100% specificity). The most significant variables in both discriminant models were the Core amino acid pattern, nHap_E1–E2, and GGT and ALT quotients. Although prediction accuracy may deteriorate in an independent sample, the internal cross-validation pointed to a better reproducibility for model 2 in a comparable population (identifying 85.7% and 81.8% of the responder and non-responder patients, respectively), despite the fact that model 1 best predicted treatment outcome in our population. Besides, using model 2 the detection of those patients likely to respond to therapy could be maximized by adjusting the cut-off, leading to a higher NPV at the cost of a lower specificity (93.3% and 63.6%, respectively, after cross-validation). Thus, the results suggest that non-response could be predicted at baseline with high accuracy (NPV after cross-validation of 81.8% to 93.3% depending on the cut-off) in patient groups comparable to ours in terms of ethnicity, clinical background, and HCV subtype.

To our knowledge, this is the first study that describes a model for predicting individual combination therapy outcomes on the basis of baseline host and viral characteristics using a discriminant multivariate analysis. This comprehensive statistical method integrates the information of all variables included in the model thus improving the prediction with respect to more commonly used statistical approaches. Additionally, discriminant models may be adjusted to include the most significant predictors of treatment outcome in each population. However, our study has several limitations: i) other viral genome regions not included in the study might also be involved in resistance to therapy, such as the ISDR. Nevertheless, a meta-analysis suggested that the association between the number of mutations in this region and SVR achievement was more pronounced in Japanese than in European patients [39]. As most European HCV-1b strains present less than 3 mutations, large sample sizes would be required to find significant associations; ii) recent studies have suggested that single nucleotide polymorphisms in several human genes involved in the IFN mediated response are associated to treatment outcome in HCV-1 infected patients, especially the *IL28B* gene polymorphisms [14]–[18]. Since our study was retrospective, whole-blood samples were not available to assess host genetic polymorphisms; iii) the sample size was limited to 43 patients. However, a similar number of patients were included in each group, accounting for the fact that about 50% of patients infected by HCV-1b achieve an SVR. Although an independent but similar population was not available, we performed an internal cross-validation. This method is commonly used to reduce classification bias and estimate future model performance [40].

Our results show that both host and viral factors are involved in treatment failure, although the exact mechanisms should be further characterized. The host-related variables included in the prediction models are routinely used for patient management and relatively easy to obtain, while viral variability estimates are obtained through laborious methods. Even so, and if confirmed in further studies, the information obtained may help physicians to restrict treatment to those patients that are likely to benefit from it, thus reducing overall treatment costs. Those patients that are unlikely to respond could avoid current therapy and related side effects, and wait for more effective treatment regimens.

In conclusion, discriminant analysis using both baseline HCV genetic variability and host characteristics has been shown as a useful statistical tool allowing us to accurately predict combination treatment outcome in a high proportion of Spanish HCV-1b infected patients. Further studies including host genetic polymorphisms and larger numbers of patients are under way, and similarly generated models will probably have an increased predictive power.

## Materials and Methods

### Ethics statement

This study was approved by the Clinical Research Ethics Committee at our institution (“Comité Ético de Investigación Clínica”, CEIC). As this was a retrospective study, and data were analyzed anonymously, informed consent was specifically waived.

### Patients and specimens

Patients with chronic hepatitis C by HCV-1b, treated with combination therapy at “Hospital Universitari Germans Trias i Pujol”, were retrospectively selected. Exclusion criteria were: previous IFN-based treatment, HIV or HBV coinfection, and having other causes of liver disease or alcohol abuse. Infection with HCV-1b was confirmed through NS5B sequencing followed by phylogenetic analysis, as previously described [41]. The patients had started antiviral therapy with PegIFN-α2a (180 µg/week) plus weight-based doses of RBV (1000–1200 mg/day) for 48 weeks between 2003 and 2008. The patients were classified into responders (patients with SVR, defined as undetectable HCV-RNA in serum 24 weeks after treatment cessation) and non-responders. Non-response was defined as continued presence of HCV-RNA during therapy (null response), rebound of HCV-RNA while on therapy (breakthrough) or 24 weeks after the end of treatment (relapse). All virological analyses were performed using serum specimens obtained before patients initiated treatment and conserved at −80°C until testing.

### Baseline clinical and epidemiological host parameters

Variables considered were gender, age, weight, source of infection, stage of fibrosis according to the Scheuer scoring system [42], and serum levels of ALT, AST, and GGT. Liver enzyme levels were transformed into a quotient expressing the factor times upper limit of normal (ULN) according to gender. We defined good treatment adherence as having received ≥80% of total maximum dose prescribed of both drugs for ≥80% of the expected duration of therapy [43].

### Baseline virological parameters

#### Serum viral load

HCV-RNA had been quantified by RT-PCR (Cobas® Amplicor HCV Monitor test, Roche Molecular Systems, Pleasanton, CA, USA) or by real-time RT-PCR (Abbott RealTi*m*e HCV assay, Abbott Molecular Inc., Des Plaines, IL, USA), according to manufacturer's instructions.

#### RNA extraction and reverse transcription (RT)

Total RNA was extracted from 220 µl of serum, using the QIAamp® viral RNA kit (QIAGEN® GmbH, Hilden, Germany) according to the manufacturer's protocol. RT was performed using random hexamers in order to prevent any bias during the reaction, as previously described [44].

#### PCR-cloning and sequencing of the E1–E2 region

A 532-bp sequence encompassing the E1 C-terminal and the E2 N-terminal regions (including the HVR-1, HVR-2 and HVR-3) was obtained and referred to as E1–E2 region (nucleotides 1322–1853 in the H77 reference sequence, GenBank accession number AF009606). PCR products were cloned and sequenced as previously described [44]. Briefly, a hemi-nested PCR was carried out with the proofreading *Pfu* DNA polymerase (Promega, Mannheim, Germany), and HCV-1b specific degenerated primers (2-Eg1 and 2-Ea, and 2-Eg2 and 2-Ea primers for the first and second rounds of PCR, respectively) [45]. Amplified DNA products were purified and cloned into *Eco*RV-digested pBluescript II SK(+) phagemid (Stratagene, La Jolla, CA, USA). Plasmids were transformed into *Escherichia coli* XL-1 blue MRF' competent cells (Stratagene). Between 25 and 35 colonies were selected and subjected to PCR followed by purification and sequencing of both strands using vector-based primers and the BigDyeTM Terminator v3.1 Ready Reaction Cycle Sequencing Kit on ABI Prism 3730 or 3100-Avant Genetic Analyzers (Applied Biosystems Foster City, CA, USA). Readings were assembled and edited with the STADEN package v1.6. [46].

#### PCR and direct sequencing of the Core region

The whole Core region (573 bp, H77 positions 342–914) was amplified using forward primer Cg1 (5′ GCCATRGTGGTCTGCGGAAC 3′, H77 positions 137–156), which was slightly modified from primer CC11 [37], and reverse primer Ca (5′ GTTGGAGCAGTCGTTCGTRA 3′, H77 positions 949–968). PCR was performed in 50 µl containing 5 µl of cDNA, 0.2 mM of each dNTP, 0.4 µM of each primer, *Pfu* buffer and 0.6 U of *Pfu* DNA polymerase (Promega). Thermocycler conditions were: 1 cycle at 94°C for 2 min, 35 cycles at 94°C for 1 min, 55°C for 2 min and 72°C for 3 min, and 1 cycle at 72°C for 7 min. PCR products were directly sequenced with the Cg2 primer (5′ GGGAGGTCTCGTAGACCGTGCAYCATG 3′, H77 positions 318–344), which was slightly modified from the Core-A1g primer [47], and the Ca primer.

#### Phylogenetic analysis of the E1–E2 region

The complete E1–E2 cloned region was subjected to phylogenetic analysis in order to rule out potential contamination between specimens and assess clustering of patients according to treatment outcome. Sequences were aligned by ClustalW implemented in MEGA 4 [48]. jModeltest [49] was used to obtain the evolutionary model that best fitted the data according to the Akaike Information Criterion. This model was employed to reconstruct a maximum-likelihood phylogenetic tree with PHYML [50]. RAxML software was used for evaluating tree reliability on the basis of branch support (1000 replicates) [51].

#### Genetic variability analysis of the E1–E2 region

Multiple alignments were generated for each patient for the complete E1–E2 region, and the HVR-1, HVR-2 and HVR-3 (H77 nucleotide positions 1491–1571, 1761–1787, and 1632–1739, respectively). The following genetic variability estimates were obtained for each multiple alignment with DnaSP v4.50 [52]: total number of polymorphic sites (S), total number of mutations (η), nucleotide diversity corrected by Jukes-Cantor method (π), and number of quasispecies variants (number of haplotypes, nHap). The number of nonsynonymous substitutions per nonsynonymous site (Ka) and Ks were obtained using the Nei-Gojobori method.

#### Amino acid composition analysis in the E1–E2 region

This analysis aimed to detect any amino acid position in the E1–E2 region that differed between groups but showed within-group homogeneity. Consensus sequences were compared between groups with the program VESPA [53] to obtain the predominant sequence for each group. The VESPA output file was employed to estimate the *G*-statistics in each amino acid position as previously described [31], where *p*-values ≤0.05 were considered significant. The false discovery rate procedure was used to correct for multiple comparisons.

#### Amino acid composition analysis of the Core region

Direct sequences obtained were analysed as described for the E1–E2 region. Sequences were also aligned to assess the presence of amino acid polymorphisms associated to treatment outcome.

#### Statistical analysis

Clinical and virological values were compared between responders and non-responders in bivariate analysis using Student's *t* test or Mann-Whitney *U* test for quantitative variables, and Chi-square or Fisher's exact tests for categorical variables. Data was expressed as mean ± standard deviation, median and range, or relative frequency. Values between 1.5 and 3 inter-quartile range above/below the upper/lower quartile of quantitative variables were identified as outliers.

Statistical models were developed to predict non-response. A multivariate logistic regression analysis was performed, where covariates included in the model were explanatory variables that achieved a *p*-value <0.20 on bivariate analyses. Variables which presented high correlations with other variables (Spearman's correlation >0.7) were also excluded to avoid colinearity problems. To obtain the final set of variables included in the model we used a backward stepwise selection procedure [54]. OR and CI_95%_ were reported for significant variables. Two discriminant analyses were also carried out [55]. In model 1 all covariates analyzed but those which presented high correlations with other variables were considered. Variables with a skewed distribution were transformed using quadratic or Log transformations and multivariate normality was tested using Henze-Zirkler's test [56]. The final discriminant function was obtained using a backward stepwise variable selection procedure. To assess how the results obtained would generalize to an independent but similar data set, each case was classified by the functions from all cases other than that case (“leave-one-out” cross-validation); this validation was performed in the whole stepwise variable selection procedure. Chi-square test was used to test the equality of covariance structures across groups [57], considering a pooled covariance matrix when the value was not significant at the 0.1 level. Model 2 included covariates that achieved a *p*-value <0.15 on bivariate analyses with the goal to improve the cross-validation results. ROC curves were obtained and the following parameters were calculated to measure the effectiveness of prediction: AUC, sensitivity (proportion of responders which are correctly identified), specificity (proportion of non-responders which are correctly identified), NPV and PPV. These parameters were also computed after cross-validation taking into account all misclassified patients in any of the 43 replications. Cut-off values that yielded highest sensitivity and specificity were selected by ROC curve analysis for the three predictive models obtained. *P*-values <0.05 were considered significant. Statistical analyses were performed using the statistical software packages SPSS v15.0 and SAS v9.1 (SAS Institute Inc., Cary, NC, USA).

#### Accession numbers

All sequences obtained in this study were submitted to the EMBL Nucleotide Sequence Database (http://www.ebi.ac.uk/embl/) under the following accession numbers: FN675941-FN675983, FN675984-FN676976, and FN676977-FN677019 for Core, E1–E2 and NS5B regions, respectively.

## Supporting Information

Figure S1 All viral sequences obtained for each patient are identified with a vertical line, the patient identification number and the response group (R, responders; NR, non-responders). Substitution model: GTR+G+I (gamma shape parameter: 0.926, proportion of invariable sites: 0.271). All nodes corresponding to each individual patient were supported with bootstrap values >70%. The scale bar represents 0.05 substitutions per nucleotide position.
(0.02 MB PDF)

Text S1 (0.03 MB DOC)
