# Supplementary figures and images for: Baseline Prediction of Combination Therapy Outcome in Hepatitis C Virus 1b Infected Patients by Discriminant Analysis Using Viral and Host Factors
Source: PLoS One. 2010 Nov 30;5(11):e14132. doi: 10.1371/journal.pone.0014132 (PMC2994723; doi:10.1371/journal.pone.0014132)

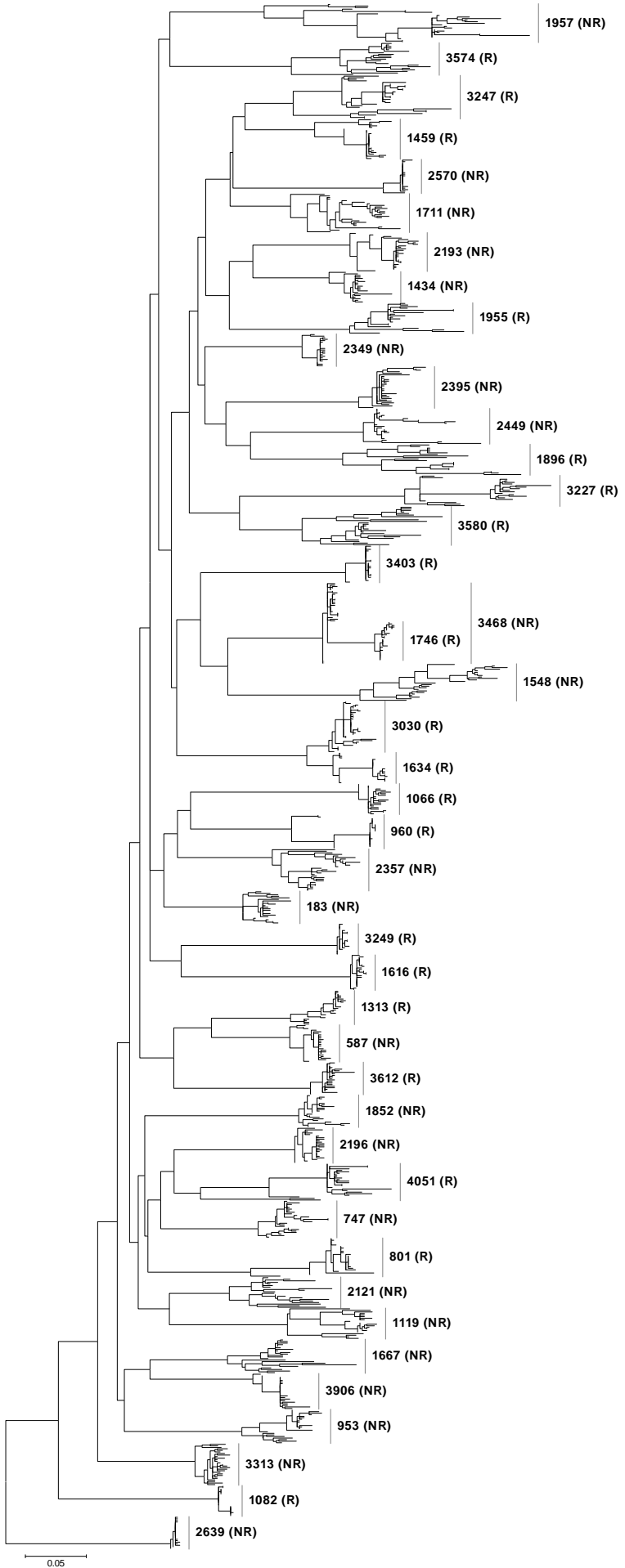

Supplement: Figure S1 — All viral sequences obtained for each patient are identified with a vertical line, the patient identification number and the response group (R, responders; NR, non-responders). Substitution model: GTR+G+I (gamma shape parameter: 0.926, proportion of invariable sites: 0.271). All nodes corresponding to each individual patient were supported with bootstrap values >70%. The scale bar represents 0.05 substitutions per nucleotide position. (0.02 MB PDF) [file pone.0014132.s001.pdf]
