# Supplementary material for: Baseline Prediction of Combination Therapy Outcome in Hepatitis C Virus 1b Infected Patients by Discriminant Analysis Using Viral and Host Factors
Source: PLoS One. 2010 Nov 30;5(11):e14132. doi: 10.1371/journal.pone.0014132 (PMC2994723; doi:10.1371/journal.pone.0014132)
Supplement: Text S1 — (0.03 MB DOC) [file pone.0014132.s002.doc]

**TEXT S1. Developed models.**

**Logistic regression model:**

$Log\left(\frac{p}{1-p}\right)$ = 0.7044 + 1.6187 × Core - 0.3875 × nHap_E1-E2

**Discriminant functions (model 1):**

f_R_ = - 95.11 + 6.834 × log_10_(viral load) + 1.644 × nHap_E1-E2 -

- 13.5 × log_10_(Ks_E1-E2) + 6.783 × Sqrt(S_HVR-2) + 17.59 × Sqrt(ALT quotient) +

+ 0.5 × weight + 2.407 × Core + 1.038 × Sqrt(GGT quotient)

f_NR_ = - 103.46 + 6.229 × log_10_(viral load) + 2.226 × nHap_E1-E2 -

- 13.55 × log_10_(Ks_E1-E2) + 6.205 × Sqrt(S_HVR-2) + 14.76 × Sqrt(ALT quotient) +

+ 0.525 × weight + 7.067 × Core + 3.432 × Sqrt(GGT quotient)

*p* = exp(f_NR_) / (exp(f_R_) + exp(f_NR_))

**Discriminant functions (model 2):**

f_R_ = - 43.85 + 2.097 × nHap_E1-E2 – 0.9787 × nHap_HVR-1 +

+ 2.384 × Sqrt(Ks_HVR-1) + 13.8 × Sqrt(ALT quotient) +

+ 1.852 × Sqrt(GGT quotient) + 6.941 × Core + 0.4953 × weight

f_NR_ = - 56.54 + 2.836 × nHap_E1-E2 – 1.466 × nHap_HVR-1 +

+ 11.28 × Sqrt(Ks_HVR-1) + 10.37 × Sqrt(ALT quotient) +

+ 4.595 × Sqrt(GGT quotient) + 11.78 × Core + 0.5173 × weight

*p* = exp(f_NR_) / (exp(f_R_) + exp(f_NR_))

**Description of variables:**

- *p*, probability of non-response.
- f_R_, value (score) on the canonical discriminant function for a case in the responder group.
- f_NR_, value on the canonical discriminant function for a case in the non-responder group.
- Core, presence of both amino acids arginine at position 70 (R70) and leucine at position 91 (L91) is coded as “0”, and absence of both R70 and L91 is coded as “1”.
- nHap, number of quasispecies variants or haplotypes.
- Viral load, serum viral load (IU/mL).
- Ks, number of synonymous substitutions per synonymous site.
- S, total number of polymorphic sites.
- ALT quotient, alanine transaminase levels (U/L) divided by the upper limit of normal for males and females (41 and 31 U/L, respectively).
- Weight, body weight (Kg).
- GGT quotient, gamma-glutamyl transferase divided by the upper limit of normal for males and females (85 and 50 U/L, respectively).

**HCV genomic regions** (nucleotide positions corresponding to the H77 reference sequence, GenBank accession number AF009606): Core, 342-914; E1-E2, 1322-1853; HVR-1, 1491-1571; HVR-2, 1761-1787.
